# Developing a bespoke patient-reported outcome measure (PROM) for genital dermatoses: A pilot service evaluation

**DOI:** 10.1177/09564624251406029

**Published:** 2025-12-08

**Authors:** Adhvika Rajesh, Eliza Maxwell, Lauma Sarkane, Jack Eaves, Beverley Azoba, Cindy Sethi

**Affiliations:** 1 Faculty of Life Sciences and Medicine, 4616 King’s College London, London, UK

**Keywords:** genital dermatoses, patient-reported outcome measures (PROMs), quality of life, sexual health

## Abstract

**Background:**

Genital dermatoses (GDs) significantly impact sexual health and psychosocial wellbeing, yet no specific patient-reported outcome measure (PROM) exists for this population. Our study aimed to appraise patient perspectives on GDs by composing a PROM to evaluate biopsychosocial concerns and compare outcomes between new and follow-up patients results.

**Methods:**

A literature review of applicable existing PROMs resulted in the selection of 14 pre-validated questions covering 3 domains: physical symptoms, quality-of-life and mental health. The response rate was 77.1% for the final PROM, with 48 responses for analysis following the exclusion of 6 incomplete responses.

**Results:**

The follow-up group showed a reduction in average scores compared to new patients, decreasing from 2.41 to 2.05 (*p* = 0.0002) with a lower score indicating better outcomes. All the questions showed a decrease in score from new to follow-up patients. Results revealed significant improvements in the average pain score among follow-up patients.

**Conclusions:**

Our findings demonstrate the necessity of PROMs in improving patient-centred care. Results indicated significant improvements in physical wellbeing in follow-up patients, affirming efficacy of existing treatments and suggested a necessity for greater psychological support to those suffering from genital dermatoses.

## Summary

Genital dermatoses significantly impact sexual health and psychosocial wellbeing, yet no specific patient-reported outcome measure (PROM) exists for this group. We developed a novel PROM by collating validated items from existing tools to assess the biopsychosocial burden of genital dermatoses in a sexual health clinic. In this pilot study, 55 patient responses were analysed. Statistically significant improvements were observed in the pain domain between new and follow-up patients. Quality of life sex life and mental wellbeing did not show significant change. This feasibility project demonstrates that PROMs can meaningfully contribute to patient-centred care and identifies areas for future refinement.

## Introduction

Genital dermatoses are common but underreported conditions affecting the genital and perianal regions, such as lichen sclerosus, lichen planus and psoriasis.^1,2^ These conditions can severely impact patients’ sexual function, mental health, and quality of life^3,4^ Understanding the patient’s perspective is crucial for effective management. Patient-Reported Outcome Measures (PROMs) are established tools that support this aim, yet no PROM specifically addresses the combined sexual and dermatological impact of genital dermatoses. This gap represents a significant limitation in providing comprehensive, patient-centred care for this overlooked population.

This project sought to develop a practical, inclusive PROM suitable for use in a genital dermatology clinic in a sexual health setting, and to pilot its feasibility and evaluate impact. Our aim was to assess whether clinical interventions improved patients’ reported outcomes and to identify domains requiring additional clinical focus.

## Methods

A literature review of validated PROMs across dermatology and sexual health domains informed item selection. Final items were chosen from SF-36, PROMIS and Skindex-29.^5–7^ Items were selected based on the following criteria: relevance to both dermatology and sexual health, non-sex-specific language, 1–5 Likert scale scoring, and validation in English. A pilot PROM survey was distributed to 6 participants at a genital dermatology clinic in a sexual health service in October 2024. Patients were instructed to answer the questionnaire solely regarding the genital dermatoses they were attending the clinic for. The questionnaire was then adjusted according to inconsistencies identified by patients. Adjustments included: adding overlooked age ranges, clarifying the wording of the new/follow-up patient question, improving formatting for greater clarity, and standardising timeframes where discrepancies existed between the original PROMs and our adapted version. The resulting bespoke PROM comprised 14 questions across three domains:

(1) Pain/Physical Symptoms (5 items)
(2) Quality of Life and Sex Life (4 items)
(3) Mental Wellbeing (5 items)

Demographic, appointment type (new or follow up patient in the genital dermatoses service) and follow-up duration (time since first seen in the genital dermatoses clinic) were also recorded. No information was collected regarding specific diagnosis or treatment.

Patients attending a specialist genital dermatology clinic within a sexual health service were invited to complete a patient-reported outcome measure (PROM) survey at the start of their consultation. All participants had been referred to the genital dermatoses (GD) service from either the walk-in sexual health clinic or other clinical services. The survey was offered to all English-speaking patients attending the GD clinic between 7th November 2024 and 6 March 2025.

Patients received an information sheet outlining the purpose of the evaluation, and participation was voluntary. Clinicians were available to answer any questions about the study during the consultation.

Data were analysed using non-parametric statistical methods, as distribution testing via the Kolmogorov–Smirnov test indicated non-normality. Differences between new and follow-up patients were compared using the Mann-Whitney U test. Statistical significance was set at *p* < 0.05.

## Results

48 responses were included in the final analysis, following the exclusion of 6 responses due to inadequate completion. The response rate of the survey was 77.1%. 28 responses (58%) were follow-up appointments and 20 (42%) were new appointments. Of the 28 participants who reported attending follow-up, 5 did not specify the duration. Among the remaining 23 participants, the mean follow-up length was 6.5 months. There were 28 male (58%) and 20 female (42%) participants, and an age range from 18 to 25 years to over 60 years. The median age group was 30-35 years.

**Figure 1.**
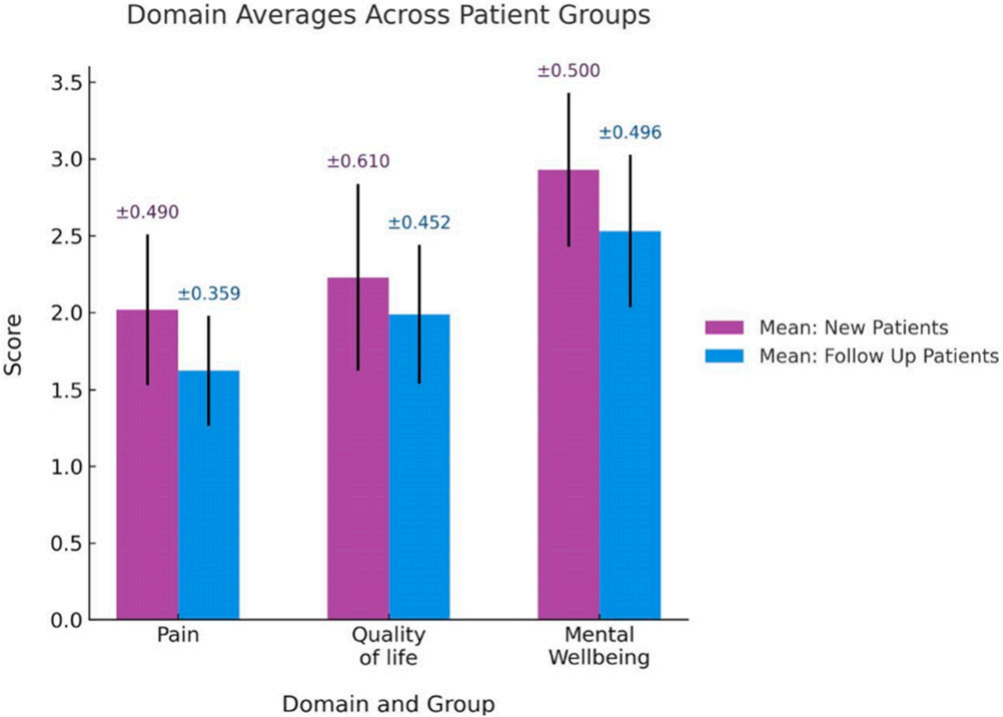
Mean Patient-Reported Outcome Measure scores across domains for new and follow-up patients. Bars represent mean scores for each group with error bars outlining 95% confidence intervals.

Ethnic representation included White (27, 56%), Black/African/Caribbean/Black British (7, 15%), Asian/Asian British (7, 15%), Mixed/Multiple ethnic groups (3, 6%), and Other (4, 8%). Follow-up patients demonstrated significantly lower overall scores compared to new patients, decreasing from 2.41 to 2.05 (*p* = 0.002), with lower scores indicating better outcomes. Statistically significant improvements were observed in the Pain/Physical Symptoms domain (*p* = 0.006). Mental wellbeing, quality of life and sex life showed an improvement but did not reach statistical significance. Domain scores are summarised in Table 1 and visualised in Figure 1.

**Table 1.** Comparison of domain scores between new and follow-up patients. *p* value significance = 0.05.

Domain	New patients mean (SD)	Follow-up patients mean (SD)	*p*-value
Pain/physical symptoms	2.02 (1.11)	1.62 (0.97)	0.006
Quality of life and sex life	2.23 (1.39)	1.99 (1.22)	0.358
Mental wellbeing	2.93 (1.15)	2.53 (1.34)	0.070
Overall average score	2.41	2.05	0.0002

## Discussion

This pilot study demonstrates the feasibility of implementing a bespoke PROM for genital dermatoses within a genital dermatoses service. Improvements in the pain domain suggest that clinical interventions are effective in addressing some key patient concerns. However, the lack of significant improvement in mental wellbeing, quality of life and sex life highlights a potential gap in current service provision and suggests that these aspects may require more targeted clinical attention.

Several limitations must be acknowledged. The small sample size precludes generalisable conclusions and required the use of non-parametric statistical methods, which have lower power to detect differences. The PROM, while composed of validated items, has not undergone formal psychometric validation in its new format, and cognitive processing differences may exist. It is also possible that comorbid systemic disease, such as psoriatic arthritis, may influence mental wellbeing and physical functioning independently of genital disease activity, potentially confounding the observed outcomes. Additionally, demographic analysis was limited by sample size heterogeneity.

Nevertheless, these preliminary findings justify further research. A larger, multi-centre study with psychometric validation of the PROM would provide stronger evidence. Furthermore, evaluation could be extended to disease- and treatment-specific analyses, such as for psoriasis, lichen sclerosus, and lichen planus. This analysis could highlight discrepancies in disease specific outcomes that require attention. Engagement with psychologists and patient focus groups could enhance future development.

In conclusion, integrating PROMs into sexual health services for patients with genital dermatoses is achievable and beneficial. Systematic patient feedback can highlight unmet needs and drive holistic improvements in care. This pilot study provides a foundation for ongoing quality improvement and patient-centred service development.
